# A measurement of the attenuation of radiation from F‐18 by a PET/MR scanner

**DOI:** 10.1002/acm2.12479

**Published:** 2018-10-19

**Authors:** Richard E. Wendt, Hua A. Ai, Joseph G. Meier, Benjamin P. Lopez, Samuel J. Fahrenholtz, Osama R. Mawlawi

**Affiliations:** ^1^ Department of Imaging Physics The University of Texas MD Anderson Cancer Center Houston Texas; ^2^ Imaging Physics Residency Program The University of Texas MD Anderson Cancer Center Houston Texas; ^3^ Graduate Program in Medical Physics The University of Texas MD Anderson Cancer Center UTHealth Graduate School of Biomedical Sciences Houston Texas; ^4^ Present address: Department of Diagnostic Radiology and Nuclear Medicine Rush University Medical Center Chicago IL USA

**Keywords:** PET/MR, self‐attenuation

## Abstract

The attenuation of 511 keV photons by the structure of a PET/MR scanner was measured prior to energizing the magnet. The exposure rate from a source of fluorine‐18 was measured in air and, with the source placed at the isocenter of the instrument, at various points outside of the scanner. In an arc from 45 to 135 degrees relative to the long axis of the scanner and at a distance of 1.5 m from the isocenter, the attenuation by the scanner is at least 5.6 half‐value layers from the MR component alone and at least 6.6 half‐value layers with the PET insert installed. This information could inform better design of the radiation shielding for PET/MR scanners.

**Abbreviations**
ALARA: as low as reasonably achievable
CT: (x‐ray) computed tomography
MR: magnetic resonance (imaging)
PET: positron emission tomography

## 1 INTRODUCTION

During the planning for the installation of a PET/MR scanner (Signa, General Electric Healthcare, Chicago, IL), no information was available on the attenuation of radiation from PET radionuclides by the scanner itself. As a consequence, the facility was shielded conservatively against ionizing radiation by ignoring any attenuation by the instrument itself. Prior to energizing the magnet during the installation of the scanner, the attenuations by the MR portion alone and by the MR portion along with the PET insert of the radiation from a source of fluorine‐18 were measured. Those measurements demonstrate that including the structure of the PET/MR scanner in a radiation shielding design could obviate an appreciable amount of structural shielding compared to that which is called for when treating the radioactive patient as a bare point source in air.

## 2 MATERIALS AND METHODS

The attenuation of the PET/MR scanner was measured both before and after the installation of the PET insert. Both measurements were made prior to the ramping up of the magnet.

A polar grid was laid out on the floor of the scanner room. The measurement points on this grid were defined at radial increments of 0.5 m from the isocenter and at azimuthal increments of 22.5 degrees in half of the room and at 45‐degree increments in the other half of the room. The measurements were made only in the horizontal plane that was at the level of the isocenter. A source of fluorine‐18 with a starting activity of 24 mCi (888 MBq) for the first set of measurements and 40 mCi (1.48 GBq) for the second set of measurements was placed at the isocenter of the scanner.

Three calibrated ionization survey meters (451B, Fluke Biomedical, Cleveland, OH) were used. Each was operated by a different medical physicist. A string with a plumb bob at the free end was tied around each meter and its length was adjusted so that the center of the chamber of the meter was in the horizontal plane of the isocenter when the tip of the plumb bob just touched the floor. Another medical physicist recorded the reading of each meter at each point on the polar grid (i.e., with the tip of the plumb bob just grazing each marked point on the floor) along with the time of day of the measurement as determined by a cellular telephone. All of the measurements were decay‐corrected to the time at which the source had been assayed. The background was negligible in all cases. The three measurements at each grid point were averaged, corrected to a distance of 1 m by the inverse square law and then normalized by the average of the in‐air measurements of the source at 1 m. They were then converted to half‐value layer values by taking their logarithms to the base 1/2 (i.e., by dividing their natural logarithms by the natural logarithm of one‐half).

An in‐house‐written computer program that the authors use for designing radiation shielding in nuclear medicine and PET was employed to estimate the weekly doses from a busy workload consisting of an average activity of 3.4 mCi (126 MBq) of F‐18 for 87.5% of the patients and an average activity of 10.2 mCi (377 MBq) of F‐18 for 12.5% of the patients. The 3.4 mCi (126 MBq) average activity while the patient is in the scanner room assumes an 11 mCi (407 MBq) administered activity, a 64% patient transmission factor, a 1 h uptake time, voiding of 15% of the administered activity, and a 1 h scanning time. The higher activity was used to simulate anticipated Zr‐89 studies. The patient was modeled as a point source that was located on the patient handling system outside of the magnet for 10% of the work week and at seven locations along the central axis of the scanner for a total of 70% of the work week, simulating the movement of the patient during image acquisition. It was assumed that the scanner room would not contain radioactivity for 20% of the work week. An occupancy factor of unity was assumed. The scanner was modeled as an annular cylinder of lead with a thickness of 33.7 mm. For 511 keV photons, this is 6.6 times the broad‐beam half‐value layer of 5.1 mm, which is the average of the values in two recent references.^1^, ^2^

## 3 RESULTS

The gamma ray exposure constant from F‐18 in syringes suspended in air was measured to be 0.573 mR‐m^2^/mCi‐hr in the first measurement session and 0.656 mR‐m^2^/mCi‐hr in the second measurement session.

**Figure 1:**
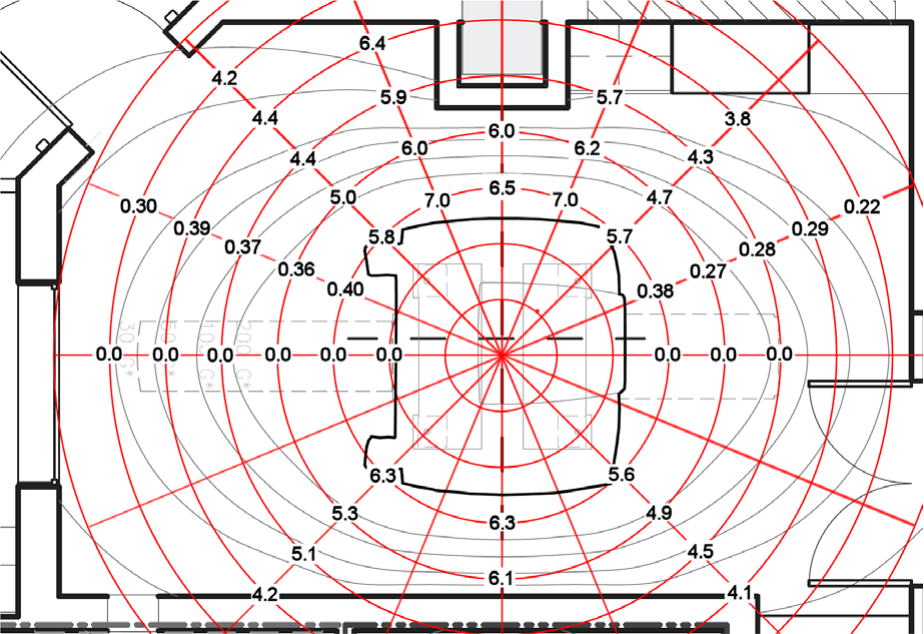
Attenuation of the MR component of the PET/MR scanner. The attenuation values in units of half‐value layer of the MR component of the PET/MR scanner are shown on the polar grid, shown in red, on which the measurements were made. The azimuthal increment of the grid was 22.5 degrees and the radial increment was 0.5 m. The outline of the MR scanner is shown in black.

**Figure 2:**
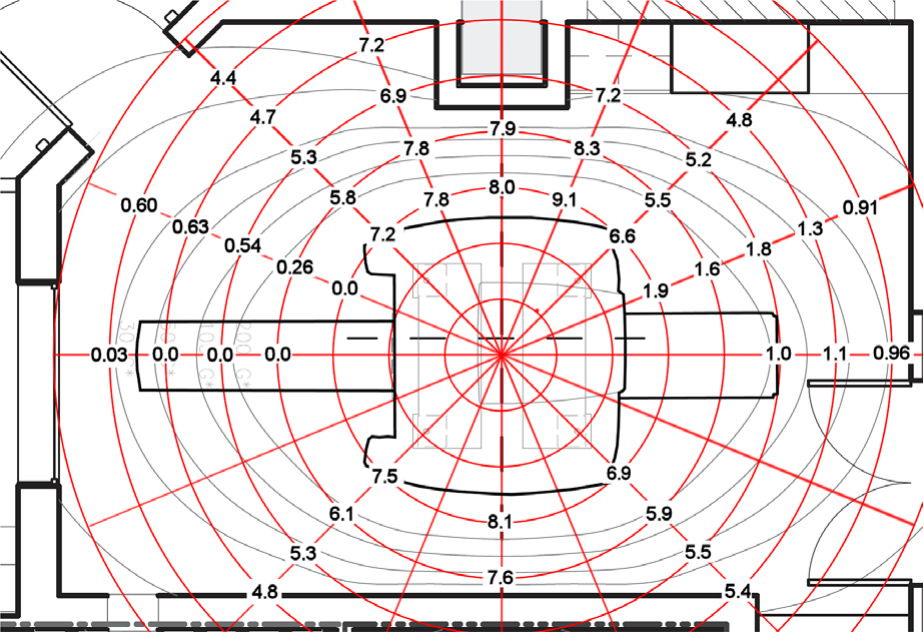
Attenuation of the MR and PET components as well as of the patient handling system of the PET/MR scanner. The attenuation values in units of half‐value layer of the scanner with the PET insert and the patient handling system installed are shown on the polar grid.

The measured half‐value layers of the attenuation afforded by the MR component alone are shown in Fig. 1 and are tabulated in Table 1. These measurements have been previously reported in a preliminary form.^3^ The measurements from the scanner after the PET insert and the patient handling system had been installed are shown in Fig. 2 and are tabulated in Table 2.

**Table 1:** Half‐value layers of the MR alone

Radial distance (m)	Azimuthal angle											
	0°	22.5°	45°	67.5°	90°	112.5°	135°	157.5°	180°	225°	270°	315°
1.5	0.0	0.40	5.8	7.0	6.5	7.0	5.7	0.38	0.0	5.6	6.3	6.3
2	0.0	0.36	5.0	6.0	6.0	6.2	4.7	0.27	0.0	4.9	6.1	5.3
2.5	0.0	0.37	4.4	5.9		5.7	4.3	0.28	0.0	4.5		5.1
3	0.0	0.39	4.4	6.4			3.8	0.29		4.1		4.2
3.5	0.0	0.30	4.2					0.22				

**Table 2:** Half‐value layers of the MR and PET insert

Radial distance (m)	Azimuthal angle											
	0°	22.5°	45°	67.5°	90°	112.5°	135°	157.5°	180°	225°	270°	315°
1.5		0.0	7.2	7.8	8.0	9.1	6.6	1.9		6.9	8.1	7.5
2	0.0	0.26	5.8	7.8	7.9	8.3	5.5	1.6		5.9	7.6	6.1
2.5	0.0	0.54	5.3	6.9		7.2	5.2	1.8	1.0	5.5		5.3
3	0.0	0.63	4.7	7.2			4.8	1.3	1.1	5.4		4.8
3.5	0.3	0.60	4.4					0.91	0.96			

**Figure 3:**
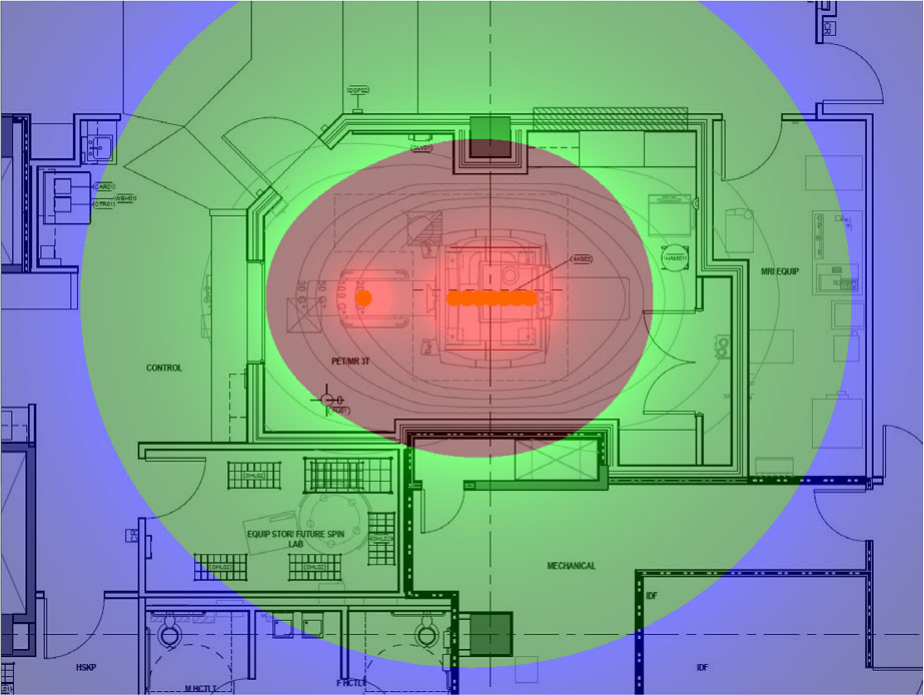
Estimated weekly dose map from bare sources simulating a busy work week. The color coding of the dose map is in shades of blue for weekly doses below 20 uSv, which is the limit for the general public in the United States; shades of green for weekly doses between 20 and 100 uSv, which is 10% of the occupational dose limit in the United States and the ALARA 1 limit of the institution in which this scanner was installed; and shades of red for weekly doses of more than 100 uSv, which would exceed the ALARA 1 threshold.

**Figure 4:**
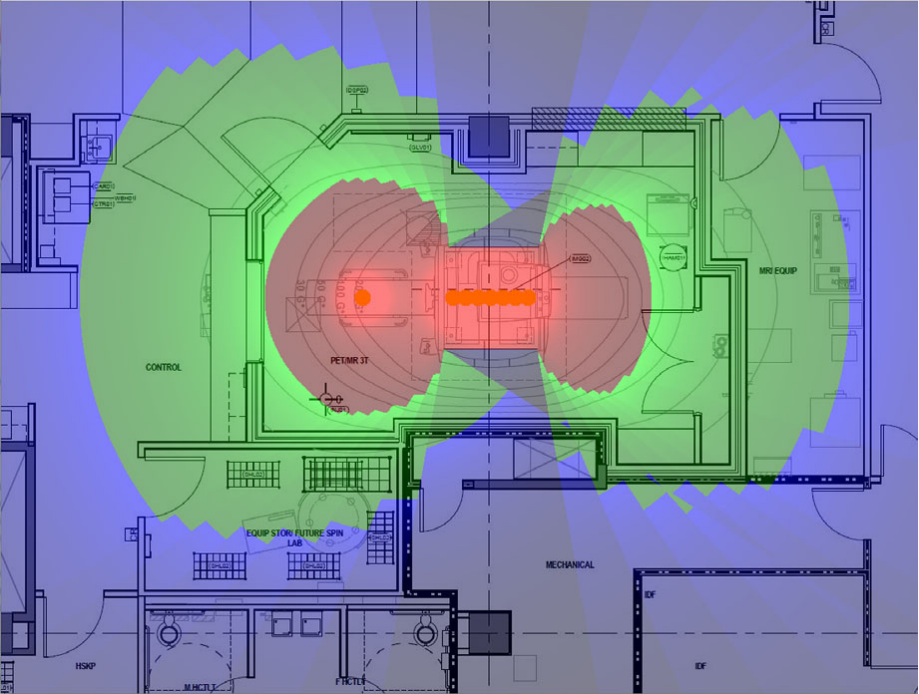
Estimated weekly dose map including attenuation by the PET/MR scanner. The red zone is entirely within the scanner room and the green zone does not extend far beyond the walls of the room. The gray areas close to the scanner are very low dose areas (i.e., a “cold” blue). When a fractional occupancy factor for members of the public is applied to the hallway at the top of the figure and to the rooms at the bottom of the figure, there is no need for any structural shielding in this particular example.

The estimated weekly dose distribution from the simulated work week in the absence of any shielding from the PET/MR itself is shown in Fig. 3. The estimated weekly dose distribution from the simulated work week including an approximation of the shielding afforded by the scanner is shown in Fig. 4.

## 4 DISCUSSION AND CONCLUSION

The gamma ray exposure constants of 0.573 and 0.656 mR‐m^2^/mCi‐hr that were measured in the two sessions differ by about 14%, which is more than the expected uncertainty in the dose calibrator readings. It is possible that the assay of the second source was not correctly performed or recorded. However, both measured constants fall within the range of values from 0.568 to 0.710 mR‐m^2^/mCi‐hr that are found in the literature.^2^, ^4^ The latter value was converted from the original dose units using a calculated f‐factor of 0.965 cGy/R based on previous studies.^5^, ^6^ This discrepancy does not adversely affect the attenuation values, which are based upon the ratio of two measurements of the same source.

Although measurements were made only in the horizontal plane that passed through the isocenter of the scanner, a visual inspection of the scanner with the covers removed indicated that that plane intersected the fewest ancillary components and that the parts such as the cold head above the magnet dewar and the supporting structure below the dewar might offer some additional shielding. The effect of extra components was observed in the horizontal plane where the patient handling system, which had been installed between the first and second measurements, introduced an additional half‐value layer of shielding along the central axis beyond it, although only over a very small solid angle.

This report complements the work of Nye, et al.,^7^ who found the effective attenuation of a General Electric Discovery LS PET/CT to be two half‐value layers along the line perpendicular to the long axis of the scanner and passing through the isocenter and of Busse^8^ who, for several PET/CT scanners, found the attenuation by a PET scanner perpendicular to the long axis to be between 1.5 and 2.3 half‐value layers, depending upon the manufacturer. The present measurements suggest that the attenuation of the PET insert of the PET/MR scanner is between 1.5 and two half‐value layers.

The structure of the PET/MR scanner itself provides a substantial amount of shielding. It is at least 6.6 half‐value layers at a distance of 1.5 m from the isocenter around an arc from 45 to 135 degrees with respect to the long axis. The thickest structural shielding that the authors have ever recommended for a PET/CT installation is five half‐value layers.

Structural shielding for PET is both heavy and expensive. When designing the installation described here, the weight of the shielding as well as that of the scanner itself was a significant concern to the structural engineers. Had this information been available then, less structural shielding would have been recommended and siting would have been easier.

## CONFLICT OF INTEREST

The authors have no conflicts of interest with respect to this work.
